# Electrochemical-Based Biosensors on Different Zinc Oxide Nanostructures: A Review

**DOI:** 10.3390/ma12182985

**Published:** 2019-09-15

**Authors:** Muhammad Luqman Mohd Napi, Suhana Mohamed Sultan, Razali Ismail, Khoo Wei How, Mohd Khairul Ahmad

**Affiliations:** 1 Computational Nanoelectronic Research Lab, School of Electrical Engineering, Universiti Teknologi Malaysia Johor Bahru, Skudai 81310, Malaysia; 2 Microelectronics and Nanotechnology-Shamsuddin Research Centre, Universiti Tun Hussein Onn Malaysia, Parit Raja 86400, Malaysia

**Keywords:** nanoparticle, nanorod, nanosheet, nanoflower, zinc oxide, electrochemical biosensor

## Abstract

Electrochemical biosensors have shown great potential in the medical diagnosis field. The performance of electrochemical biosensors depends on the sensing materials used. ZnO nanostructures play important roles as the active sites where biological events occur, subsequently defining the sensitivity and stability of the device. ZnO nanostructures have been synthesized into four different dimensional formations, which are zero dimensional (nanoparticles and quantum dots), one dimensional (nanorods, nanotubes, nanofibers, and nanowires), two dimensional (nanosheets, nanoflakes, nanodiscs, and nanowalls) and three dimensional (hollow spheres and nanoflowers). The zero-dimensional nanostructures could be utilized for creating more active sites with a larger surface area. Meanwhile, one-dimensional nanostructures provide a direct and stable pathway for rapid electron transport. Two-dimensional nanostructures possess a unique polar surface for enhancing the immobilization process. Finally, three-dimensional nanostructures create extra surface area because of their geometric volume. The sensing performance of each of these morphologies toward the bio-analyte level makes ZnO nanostructures a suitable candidate to be applied as active sites in electrochemical biosensors for medical diagnostic purposes. This review highlights recent advances in various dimensions of ZnO nanostructures towards electrochemical biosensor applications.

## 1. Introduction

For the past decade, zinc oxide (ZnO) biosensors have become the prevalent topic in thin film research areas. Distinctive features of ZnO, such as good electrical transport, wide band gap, and high exciton binding energy of 3.37 eV and 60 meV respectively, has shown potential as a new material for biosensing applications [1,2,3,4,5]. The wide band gap has the ability to sustain large electric fields, which allow a high breakdown voltage and stable semiconductor in the visible region [6]. ZnO has been recognized as a good candidate for biosensor applications because of its high isoelectric point (IEP), cost effectiveness, nontoxicity, and chemical stability [1,3,7]. A high value of IEP allows a better absorption process of enzymes, DNA, and proteins by electrostatic interactions [7]. ZnO has been used widely to detect various species such as glucose [8], cholesterol [7], uric acid [9], *Leptospira* [10], ascorbic acid [11], and cancer cells [12].

**Figure 1:**
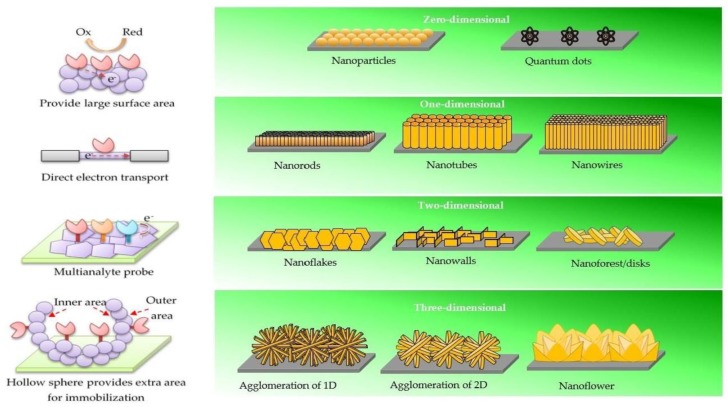
Four different dimensions of ZnO nanostructures with their advantages. 0-D nanostructures provide large surface area. 1-D nanostructures possess stable and direct electron transport. 2-D nanostructures give specific planes for immobilization process for the simultaneous detection of different analytes. 3-D nanostructures have extra surface area (outer and inner area) to provide more sites for immobilization.

ZnO is an important multifunctional nanomaterial with a number of analytical detection methods such as in mass-based biosensors [13] as well as electrochemical [14] and optical [15] methods. Among them, ZnO-based electrochemical biosensors have attracted considerable attention in healthcare applications that could significantly enhance early diagnostic capabilities with fast response times. In order to enhance the performance of the biosensor (detection limit, sensitivity, and response time), nanostructures are the most suitable material for further study. Nanostructured ZnO can be categorized into four types of dimensions, which are zero dimensional (0-D), one dimensional (1-D), two dimensional (2-D), and three dimensional (3-D), which are illustrated in Figure 1. Briefly, 0-D ZnO is the basic, elemental unit that consists of nanoparticles [16] and quantum dots [17]. 1-D ZnO corresponds to linear geometric shapes such as nanorods [18], nanotubes [19], nanowires [20], and nanofibers [21]. When the crystal has grown along the lateral direction, the surface geometric known as 2-D is formed, which basically consists of nanodisks [22], nanoflakes [23], nanosheets [24] and nanowalls [25]. The 0-D, 1-D, and 2-D classes can be agglomerated into a spherical 3-D shape that corresponds to a geometric shape with volume [26].

So far, in vivo sensing applications have largely driven the development of 0-D ZnO nanostructures [27]. Furthermore, what made it possible to immobilize various types of biomolecules successfully is the advantage of the large surface area of ZnO nanoparticles, which increases when the grain size is reduced [28,29,30]. However, the grain size of ZnO nanostructures affect carrier mobility. Small grain sizes lead to a reduced mobility of the carrier inside the materials because of the high number of grain boundaries [31]. Alternatively, 1-D ZnO nanostructures, such as ZnO nanorods and nanowires (diameter <40 nm [32]), have been utilized primarily for enhancing the electron transport performance and maintaining an oxide-stable surface [27,33,34]. Further progress of the ZnO nanostructures has been assembled from 1-D to 2-D ZnO nanostructures (nanosheets and nanoflakes), which demonstrate a high surface charge density at a specific plane inside the nanomaterial [34]. Finally, a combination of 0-D, 1-D, and 2-D subunits produced 3-D ZnO nanostructures with the volume geometric shape, has generated great interest as the active sites [35]. These hierarchical structures have shown impressive progress in sensing applications. For instance, urchin-like ZnO nanostructures possess narrower band gaps, higher donors, and fewer acceptors, thus exhibiting a better gas-sensing performance compared to ZnO nanoparticles [36]. ZnO nanotrees have indicated good responses at low concentrations and chemical stability towards heavy hydrocarbon sensing applications [37]. Currently, all of these have lacked attention in biosensing applications. Considerable attention has been paid on the deposition methods since various deposition parameters could be tailored to produce a variety of morphologies of ZnO. Nanostructures can be obtained from techniques such as spray pyrolysis deposition, hydrothermal methods, chemical vapor deposition, radio frequency sputter, pulsed laser deposition, contact printing, microwave irradiation, and inkjet printing [33,38,39,40,41,42,43].

The focus of this paper is to comprehensively report on recent progress of ZnO electrochemical biosensors based on its dimensional classes. This paper discusses the utilization of various dimensions of ZnO material, the effect of their morphology on different biomolecule detection applications, and comparisons of the sensing performances with respect to their different morphologies, and we will highlight present challenges.

### ZnO-Based Electrochemical Biosensors

**Figure 2:**
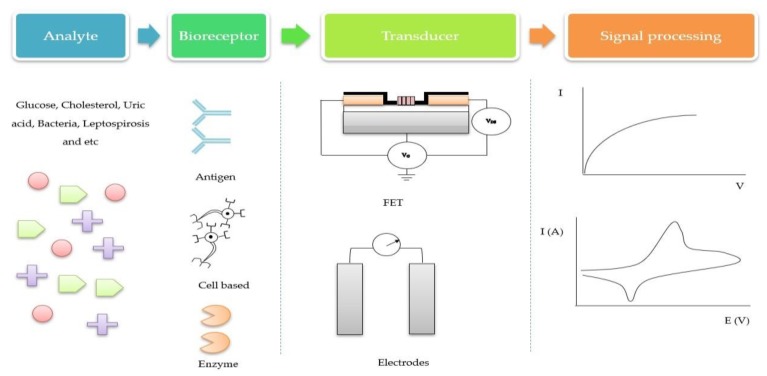
Illustration of an electrochemical biosensor system.

Over the past years, several device concepts have been developed for medical diagnostic applications. The difficulty of connecting an electronic device directly to a biological environment becomes a challenge, as biological information is not easily converted to an electronic signal. Thus, electrochemical biosensors are advantageous over other analytical methods because of their direct conversion of biological events to an electronic signal with great stability. Figure 2 illustrates the four major parts related in an electrochemical biosensor operation. In the first part, the analyte is the target element. ZnO-based electrochemical biosensors are effectively used to detect a large variety of analytes such as glucose, uric acid, cholesterol, DNA, and dopamine. In the second part, the bioreceptor is the biological recognition component that is immobilized on the active site (ZnO) platform, which has a particular binding affinity to interact with the specific analyte sample. Typical recognition components used in the device are enzymes, DNA, proteins, and antigens. Third, the transducer element provides a platform for a specific biological event to take place. The function of the transducer can be clarified as a device that converts the chemical, physical, and biological effects into an electrical signal with high sensitivity and minimum disturbance [44]. Fourth, signals are processed by computer software into meaningful physical parameters.

Typically, in electrochemistry biosensors, when an exposed active site is immobilized with an analyte, it would either produce a quantifiable current (amperometric), a quantifiable resistance and reactance (impedimetric), or measure the conductive behaviors of the ambience between electrodes (conductometric). A few surveys have also been conducted on other types of electrochemical detection approaches such as potentiometrics, which measure charge accumulation or potential. All of these measurement techniques will be summarized here. Since reactions are generally detected only in close proximity to the active site surface, the electrodes themselves play a crucial role in the performance of electrochemical biosensors.

Basically, the electrochemical biosensor system consists of three major electrodes known as sensing or redox electrodes. These electrodes are the working electrode, counter electrode, and reference electrode. During the biochemical reaction process, the working electrode serves as the transduction element. Meanwhile, current is applied to the working electrode by the counter electrode as it establishes a connection to the electrolytic solution. In order to maintain a stable potential, the reference electrode is kept at a distance from the reaction site. Normally, the reference electrode is made from Ag/AgCl. These electrodes should be both conductive and chemically stable.

The field effect transistor is based on surface conductance, which has potentiometric effects at the gate electrode [45]. FET-based biosensors are known to be the best method among others. Charge accumulation on the nanomaterial channel between source and drain electrodes results in less time to analyze the different analytes with great sensitivity and high selectivity [46,47,48]. In addition, an excellent review of the properties, such as sensitivity and fabrication process for electrochemical biosensor, are summarized by Grieshaber et al. [49] and Ahmad et al. [50].

## 2. Biosensors with Different ZnO Morphologies

### 2.1. Zero-Dimensional ZnO Nanostructures (0-D ZnO)

**Figure 3:**
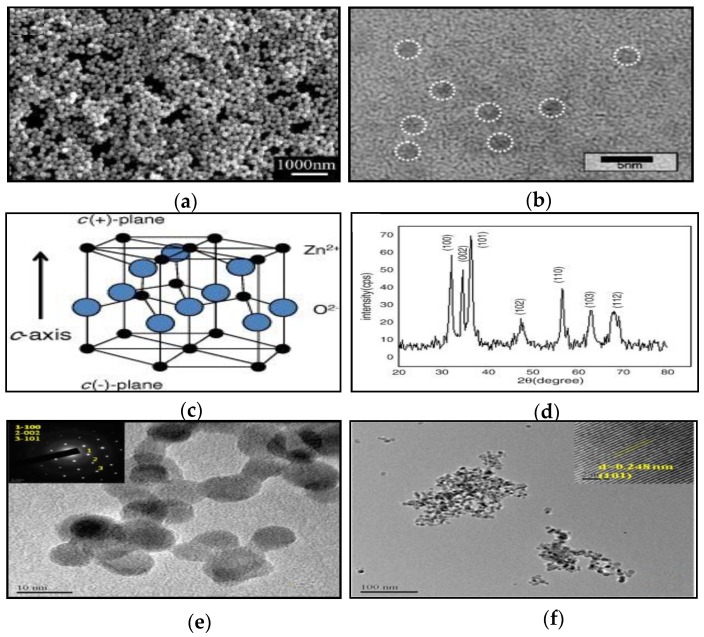
(**a**) Field emission scanning electron microscopy (FESEM) image of ZnO nanoparticles. Reprinted from [56]. Copyright 2009, with permission from Elsevier. (**b**) Transmission electron microscopy (TEM) image of ZnO quantum dots. The dark areas inside the white dashed circles correspond to the ZnO quantum dots. Reprinted by permission from Springer Nature [60]. Copyright 2008. (**c**) Schematic diagram of wurtzite structure of ZnO. Reprinted from [61]. Copyright 2016, with permission from Elsevier. (**d**) XRD peaks of polycrystalline ZnO nanoparticles. Reprinted from [62]. Copyright 2015, with permission from Elsevier. (**e**) HRTEM images with a 10 nm scale bar (inset: selected area electron diffraction (SAED) patterns indicate that the concentric circles of three main peaks are well indexed to (100), (002), and (101)). Reprinted from [52], Copyright 2017, with permission from Elsevier. (**f**) HRTEM image with 100 nm magnification of ZnO nanoparticles with 0.248 nm of lattice spacing (inset: interplanar spacing) that correspond to the d-spacing of (101) planes. Reprinted from [52] Copyright 2017, with permission from Elsevier.

The basic surface morphology of ZnO is zero dimensional (nanoparticles and quantum dots), as shown in Figure 3a,b. Figure 3c shows an illustration of the wurtzite phase of the ZnO material. From the illustration of the wurtzite structure, Noriko et al. mentioned that along the *c*-axis of ZnO structures there was spontaneous electrical polarization, due to rich zinc and oxygen, and this surface electronic polarity phenomenon would affect various properties of ZnO [51]. Figure 3d shows the formation of the wurtzite phase of ZnO observed in X-ray diffraction (XRD) patterns. The three main peaks, (100), (002), and (101), observed from XRD measurements demonstrate that 0-D ZnO nanostructures exhibit wurtzite phases. Similarly, Dayakar et al. [48] characterized their ZnO nanoparticles by high-resolution transmission electron microscopy (HRTEM) with 10 and 100 nm magnifications, as shown in Figure 3e,f, respectively. From HRTEM diffraction analyses, the selected area electron diffraction (SAED) pattern clearly indicates concentric circles of three main peaks, which are indexed to (100), (002), and (101) planes. This suggests the formation of high crystalline wurtzite hexagonal ZnO nanoparticles, which are also shown in inset Figure 3e [52].

Zero-dimensional ZnO nanostructure-based electrochemical biosensors exhibit a large potential to detect bio-element targets and have demonstrated positive results during sensing measurements. Gunjan et al. classified two vital roles of ZnO nanoparticles in biosensor applications, which are (1) size and (2) large surface-area-to-volume ratio [53]. One of the key features of ZnO nanoparticles is its size. It is considered to be a good property for medical biosensors if the size of ZnO nanoparticles is in the range of 10–100 nm [53]. As a result, 0-D ZnO is moving towards the trend of miniaturization. Furthermore, the small size of nanoparticles increases their surface area. This is beneficial for surface binding and reactivity of the sensing performance [54]. Hence, more interest has been shown towards physical and chemical properties of the materials in their nano size form (especially nanoparticles) than the solid bulk counterpart [55]. Due to this reason, ZnO nanoparticles have been used with various transduction groups, which will be discussed later.

**Figure 4:**
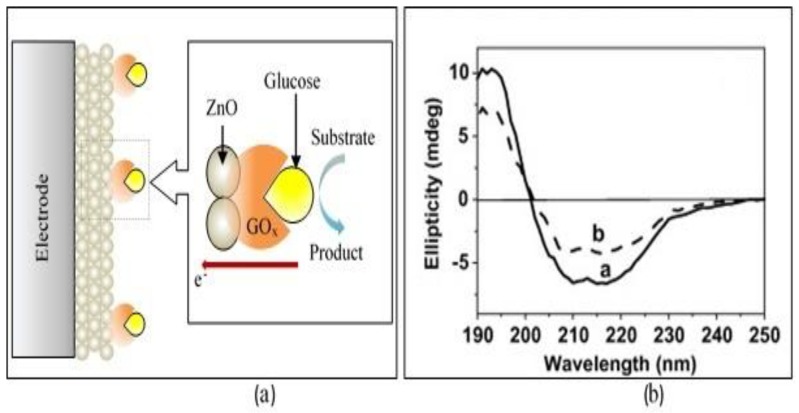
(**a**) Illustration of ZnO nanoparticles between electrode and glucose oxidase adsorbed on the nanoparticle surface. (**b**) Circular dichroism spectrum of glucose oxidase (curve a: GOx/ZnO bio-conjunction and curve b: pure GOx). Reprinted from [56]. Copyright 2009, with permission from Elsevier.

ZnO nanoparticles have been used to solve a few problems in electrochemical biosensors. Xing et al. reported that by using an enzyme as a bioreceptor, a barrier was formed for electrons to transport from the enzyme layer to the conductive electrode material because the active site of the enzyme was actually placed inside the protein shell [56]. Thus, ZnO nanoparticles were used as sandwiches between the enzyme (GOx) and electrode, as shown in Figure 4a. The small dimension of ZnO nanoparticles facilitated the functionalization of the enzyme and, as a result, improved the performance of the biosensor system due to efficient adsorption of the enzyme onto the surface of ZnO nanoparticles [56]. The adsorption of enzyme onto 0-D ZnO was confirmed from circular dichroism (CD) measurements by Ren et al. [56] to characterize the secondary structure of GOx before and after bioconjugation with ZnO nanoparticles. Figure 4b shows that the intensity of bioconjugated GOx/ZnO (curve a) is lower compared to the pure GOx spectrum (curve b), indicating that GOx successfully adsorbed on the surface of ZnO nanoparticles.

In addition, ZnO nanoparticles provide a conductive path from the enzyme to the electrode and, subsequently, improve the electric conductivity of the enzyme electrode. It accelerated the electron transfer rate between GOx and the electrode and improved the current response markedly compared to the electrode without ZnO nanoparticles. A similar trend has been observed in another work by Fidal et al. [57]. Presumably, the small grain size of ZnO nanoparticles led to an increase in the surface area. Hence, these 0-D nanostructures were able to provide many sites for enzyme immobilization processes on its surface and reduce the barriers for mass transportation during the reaction between the analyte and enzyme [58].

Recently, Aini et al. [59] fabricated an electrochemical biosensor based on glucose oxidase, ZnO nanoparticles, ionic liquid, and an eggshell membrane onto a glassy carbon electrode. They conducted a study addressing how the dimensions of ZnO nanoparticles affect their efficiency of direct electron transfer. Using ZnO nanoparticles sandwiched between eggshell membranes (as artificial membrane) and in ionic liquid improved the consignment boundary for enzyme immobilization, thus facilitating the intimate precursory interaction between glucose oxidase and the electrode.

**Figure 5:**
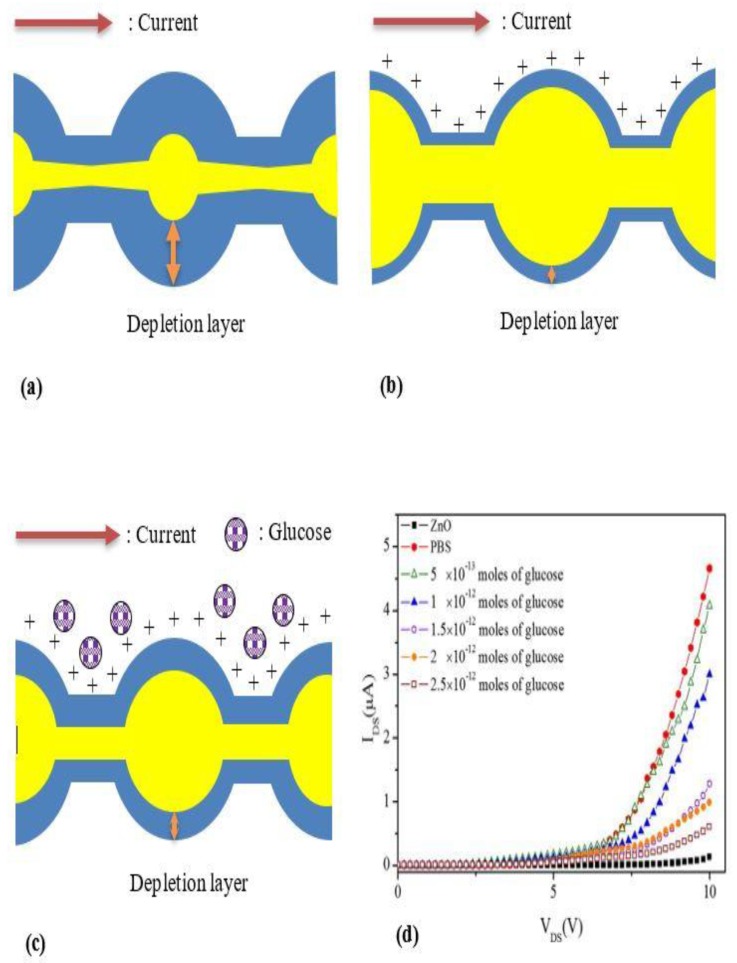
Redrawing of the sensing system of glucose concentration directly using ZnO nanoparticles (**a**) early condition of ZnO nanoparticles channel, (**b**) reduction of the depletion layer of the ZnO nanoparticles channel after immersion in phosphate-buffered saline solution, (**c**) increase of the depletion layer in the ZnO nanoparticles channel with phosphate-buffered saline after addition with glucose solution, and (**d**) current–voltage plot for glucose biosensors with different concentrations of glucose show that current value is inversely proportional to glucose concentration. Reprinted from [65]. Copyright 2011, with permission from Elsevier.

Enzymatic electrochemical biosensors have been the focus of most previous works particularly because of their specificity and responsiveness in biological environments. However, implanting the enzymatic sensor into the human body over a long term is challenging as the enzyme will degrade quickly [63]. Recently, nonenzymatic glucose electrochemical sensors have become an alternative method to overcome the disadvantages of enzymes [64]. A nonenzymatic glucose sensor was developed by Park et al., and this was the first time ZnO nanoparticles were successfully deposited on a plastic substrate [65]. ZnO nanoparticles themselves had the ability to respond (electrical characteristics) directly when exposed to the glucose solution. The mechanism of detection can be seen in Figure 5a, which shows the depletion region of the initial channel composed of ZnO nanoparticles with its respective flow of current. Then, when the phosphate buffer solution (PBS) was added into the device, the depletion layer inside the ZnO nanoparticles narrowed due to the positive charge from (PBS) around the channel surface, as shown in Figure 5b. As a result, the current transported in the channel increased. When the channel was exposed to the target analyte, the depletion layer was extended (Figure 5c) due to free electrons released by glucose, and this depletion layer was wider than as shown in Figure 5b. Consequently, the magnitude of the current falls to that of the initial channel in proportion to the number of glucose molecules. As illustrated in Figure 5d, the lowest concentration of glucose has the highest current value; thus, it can be concluded that the glucose concentration is inversely proportional to the current response.

Dayakar et al. [52] used a new environmentally friendly approach to fabricate ZnO nanoparticles using leaf extracts of *Ocimum tenuiflorum* for the highly sensitive detection of glucose without an enzymatic receptor. Because of lower toxicity, the homogenous surface, and the decreased particle size from the environmentally friendly synthesis method, ZnO nanoparticles had excellent catalytic activity in glucose oxidation. Furthermore, in a recent report by Mahmoud et al. [66], the sensitivity and catalytic activity towards glucose oxidase was improved after doping with copper (Cu). Cu-doped ZnO enhanced the electrochemical properties of nonenzymatic glucose sensors due to the presence of many electro-active sites.

As discussed in glucose detection above, the characteristic of a large surface area that leads to a high adsorption ability is worth highlighting. In addition, this dimension provides a better architecture for the immobilization of enzymes or hybrids with other biomaterials. As cholesterol has been linked to damaged arteries and leads to cardiovascular disease, ZnO nanoparticles were used as biosensors to detect cholesterol. It may be clearly inferred from previous findings in cholesterol sensing [30,67,68,69] that ZnO nanoparticles can be used as a hybrid material with other nanomaterials and electrodes.

Hayat et al. discovered (nanohybrid process) that the nanohybrid of ZnO with a carbon nanotube (CNT) could also be a potential candidate for material substitution of natural enzymes [69]. As the catalytic activities of two different materials (ZnO and CNT) were combined, with their catalytic features being retained, the produced nanocomposites proved to have an enhanced catalytic response [69] and was in good agreement with other works [29].

**Figure 6:**
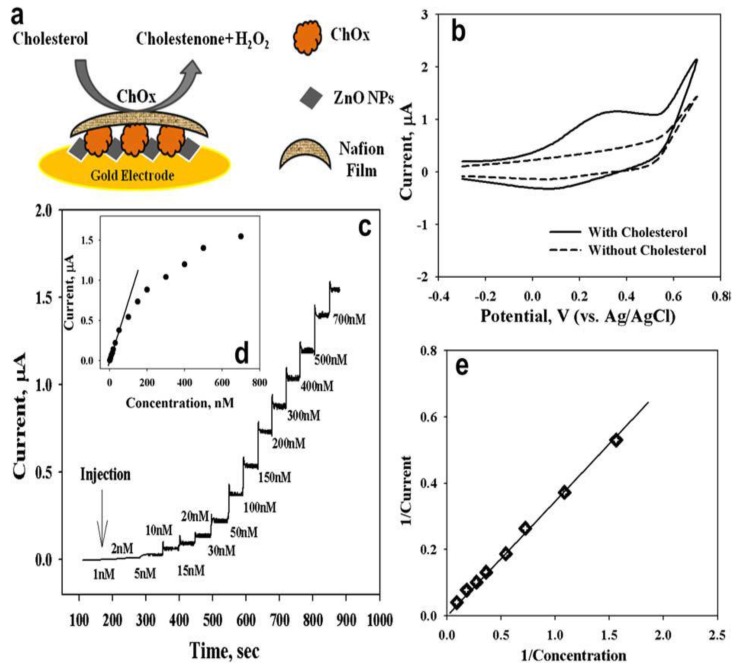
(**a**) Illustration of the cholesterol-sensing mechanism of conductive electrode/ZnO nanoparticles/Chox/Nafion(modified transducer) for enhancing their biosensor performance; (**b**) electrochemical analysis based on current vs. voltage plot for the modified transducer, solid line refers to the system exposed to cholesterol, and the dotted line without cholesterol, in phosphate-buffered saline solution; (**c**) current response time of the modified transducer when exposed to cholesterol with addition of phosphate-buffered saline; (**d**) graph curve for different cholesterol concentrations as a function of magnitude of current using a modified transducer; and (**e**) the plot of 1/current vs. 1/concentration, where a direct relationship with the cholesterol concentration and steady-state current is shown. Reprinted from [70]. Copyright 2009, with permission from Elsevier.

There was a case to fabricate amperometric biosensors for cholesterol detection by using ZnO nanoparticles as the bridge between enzyme and electrode without integrating it with other nanomaterials (Figure 6a) [70]. As a result, an effective sensitivity and low detection limit of the biosensor was recorded (refer Figure 6b–d). The adsorption of Chox (enzyme) on the ZnO nanoparticles surface allowed for easier electron transport between the active sites of functionalized Chox and solid electrode [70]. This study had good agreement with Vilian et al. [71], which showed that ZnO nanoparticles improved the electron transport between the enzyme and the electrode. Furthermore, it has been mentioned that larger surface area of 0-D has the ability to load a maximum amount of enzyme attached on its surface. This was proved by the calculation of the Michaelis–Menten constant (*K_m_^app^)* by analyzing the slope and intercept for the plot of the reciprocals of steady state current (1/I) versus cholesterol concentration (1/C) in Figure 6e. The lower value of *K_m_^app^* (4.7 mM) confirmed that the large surface area of ZnO nanoparticles had a vital role in providing efficient enzyme loading on its surface [70].

By considering the benefit of the small size of ZnO nanoparticles, Zhang et al. found that uric acid detection using nanostructures had the ability to facilitate electron transport and enhanced the adsorption strength of the biomolecule [72]. This is because the existence of ZnO nanoparticles (nanohybrid with graphene on glassy carbon electrode) could increase the electroactive surface area (EASA), which is calculated using the Randles–Sevcik equation [73]. Subsequently, it improved the sensitivity in uric acid detection [72].

Recently, Rahmanian et al. [74] fabricated a new impedimetric biosensor based on electrodeposited porous ZnO nanoparticles–polymer hybrid by covalent immobilization of enzyme for urea detection. Polyvinyl alcohol (PVA) was used to provide the porous layer with hydroxyl ions groups as the immobilization site. This polymeric soluble additive (PVA) supplies a high electronic density to the porous ZnO nanoparticles–polymer surface by free hydroxyl ions, which serves as an electrostatic repulsion layer for the anionic interferents in the bio-electrochemical medium. The porous ZnO nanoparticles–polymer successfully improved the enzyme electrode performance (minimum loss of enzyme activity) as its large surface area provided a better access to urea.

0-D ZnO quantum dots were also used to detect carbohydrate antigen 19-9 (CA 19-9), which is a preferred label for pancreatic cancer [17]. This study showed that 0-D ZnO nanostructures could be utilized for creating more active sites with large surface areas for the detection of various analytes, for example: nanohybrid ZnO and reduced graphene oxide for applications in ascorbic acid and dopamine sensing [72], ZnO nanoparticles and gold (Au) for carcinoembryonic antigen detection [75], graphene/carbon nanotube-ZnO nanoparticles for organophosporus sensors [68], ZnO nanoparticles–carboxylic graphene for acetylcholinesterase sensors [76], and ZnO nanoparticles–multiwall carbon nanotubes for lactate sensors [77]. In cancer research, biomarker detection has attracted attention with the use of nanohybrid materials using ZnO quantum dots [78,79,80]. Table 1 summarizes the 0-D ZnO-based electrochemical biosensor research conducted since 2014.

### 2.2. One-Dimensional ZnO Nanostructures (1-D ZnO)

**Figure 7:**
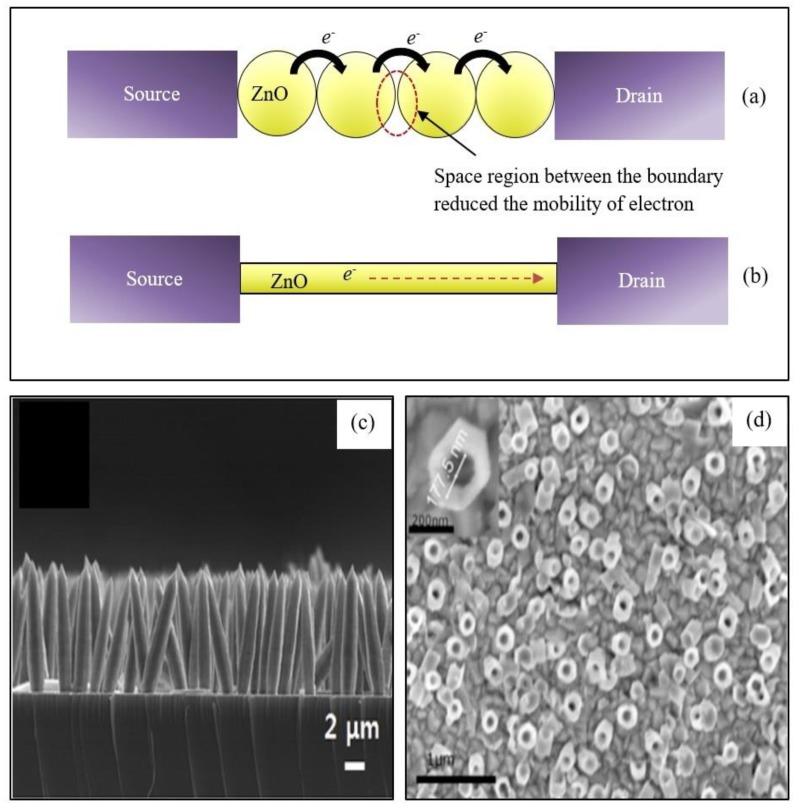
Schematic diagram for electron flow through ZnO nanostructure bridges between source and drain for (**a**) nanoparticles and (**b**) single lateral nanowire. (**c**) Cross-sectional image of ZnO nanorods. Reprinted from [94]. Copyright 2014, with permission from Elsevier. (**d**) Top view FESEM image of ZnO nanotubes. Reprinted from [19]. Copyright 2018, with permission from Elsevier.

Though 0-D ZnO nanostructures have demonstrated enhanced sensing behaviors, there are some drawbacks, as reported by some literature [91,92]. The major drawback is related to low mobility due to the high number of grain boundaries in the nanoparticles. Electron flow from terminal to terminal faces more space charge region, thus reducing the carrier mobility as illustrated in Figure 7a. Therefore, development of new dimensional nanostructures for biosensor devices has drawn increased attention. This led to the finding of one-dimensional nanostructured ZnO (1-D ZnO). This morphological group consists of various members such as nanorods (Figure 7c), nanowires, nanofibers, and nanotubes (Figure 7d). 1-D ZnO provides unique sensing advantages over 0-D ZnO. 1-D ZnO boasts high surface-to-volume ratio, and provides a direct and stable pathway for rapid electron transport [93].

**Figure 8:**
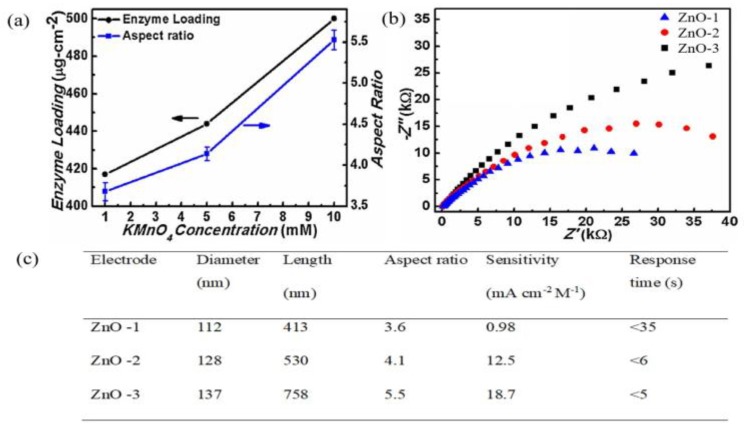
(**a**) Enzyme loading per unit area of electrode (left Y-axis) and aspect ratio variation (Right Y-axis) with variation in KMnO_4_ (mM). (**b**) The Nyquist plot of different electrodes. (**c**) Comparison of aspect ratio and sensor performance of different types of electrodes with average length and diameter distribution. Reprinted from [96]. Copyright 2017, with permission from Elsevier.

One important feature of 1-D ZnO in biosensor applications is its high aspect ratio. Demes et al. proposed that a high aspect ratio with a length above 2 µm and a diameter below 50 nm is preferable to ensure good cohesion and mechanical robustness of ZnO nanostructures [95]. Shukla et al. [96] demonstrated that 1-D ZnO with a high aspect ratio led to maximum enzyme loading, as depicted in Figure 8a [96]. In addition, charge transfer resistance value decreases significantly with 97 kΩ (ZnO-1), 55 kΩ (ZnO-2), and 40 kΩ (ZnO-3), as depicted by the Nyquist plot shown in Figure 8b. The measured results revealed that, apart from maximum enzyme loading, electron transfer characteristics were also hold a key merit of by high aspect ratio in 1-D ZnO. As a result, maximal sensitivity was recorded for ZnO-3 with a fast response time of >5 s (Figure 8c).

**Figure 9:**
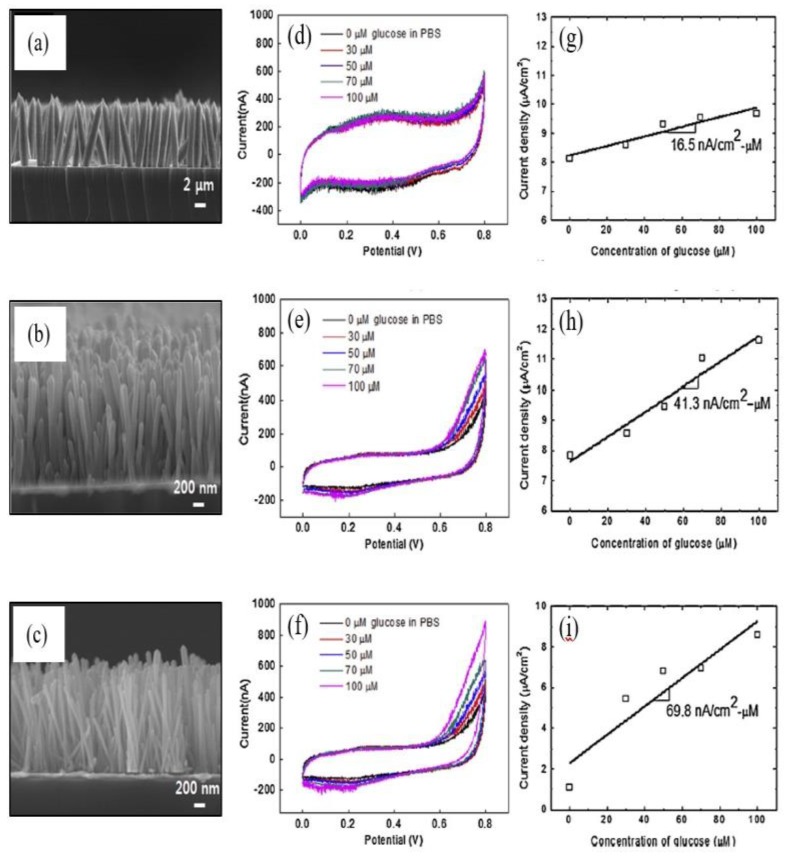
FESEM image of 1-D ZnO (**a**) ZnO-a (Length 7.7 µm; Diameter 1.5 µm); (**b**) ZnO-b (Length 1.58 µm; Diameter 0.105 µm); and (**c**) ZnO-b (Length 1.4 µm; Diameter 0.06 µm). Cyclic voltammograms of the 1-D ZnO amperometric-based glucose sensor (**d**) ZnO-a; (**e**) ZnO-b; and (**f**) ZnO-c. The current density versus glucose concentration (**g**) ZnO-a; (**h**) ZnO-b; and (**i**) ZnO-c. The slopes indicate the sensitivity of the fabricated sensors. Reprinted from Ref. [94]. Copyright 2014, with permission from Elsevier.

Apart from a high aspect ratio, Kim et al. revealed that a densely packed 1-D ZnO array is more favorable for obtaining superior ZnO-based glucose sensors, as depicted in Figure 9 [94]. In this work, the three different surface areas of 1-D ZnO (ZnO-a, ZnO-b, and ZnO-c) strongly influenced glucose sensitivity measurements. The sensitivity of low densely packed (ZnO-a), medium densely packed (ZnO-b), and high densely packed (ZnO-c) were 16.5, 41.3, and 69.8 nA/(µM cm^2^), respectively.

Another member of 1-D ZnO is nanotube structure (NTs). These NTs have been greatly used in biosensor applications due to large specific surface areas and hollow interiors instead of providing a direct electron transport pathway [97], thus making it more advanced compared to other members of 1-D ZnO. For example, Wang et al. [19] used ZnO NTs supported by molecularly imprinted polymer (MIP) arrays as electrochemical sensors for dopamine (DA) detection. MIPs are believed to alleviate selectivity problems between DA with ascorbic acid and uric acid. Interestingly, in calculations made by Wang et al. [19] using the Randles–Sevcik equation, ZnO NTs supported by MIPs had a higher active surface area of 0.2674 cm^2^ compared to nanorod structures (NRs) supported by MIPs, which was only 0.1387 cm^2^. Zhou et al.’s study found similar results, where ZnO NT-based amperometric glucose biosensors had a better sensing performance than the ZnO NRs [8]. For the first time, ZnO NTs have been used in cholesterol detection with excellent sensitivity and fast response time of 79.40 µA/mM/cm^2^ and ~2 s, respectively [98].

For further advancement in 1-D ZnO-based electrochemical biosensors for glucose detection, Zhou et al. found that hybrids with other nanoparticles (Pt, Ni, and Co) and doped by metal (Au, C, and Al) improved the catalytic activity and conductivity of ZnO [18]. This was because these glucose sensors improved the catalytic behavior and enabled the generation and transfer of GOx to the ZnO surface. Unfortunately, the low electron transfer ability of base ZnO resulted in some electrons being transferred back to the GOx active site. Thus, Zhou et al. [18] improved the transfer of redox electrons from 1-D ZnO to electrode sensors using Ag doping, resulting in a sensitivity of 85 µA/(mM·cm^2^), detection limit of 1.5 µM, and linear range of 1.5 × 10^−3^ to 6.5 mM.

Doping method on 1-D ZnO have been also used in malaria detection, as proposed by Paul et al. [99], by introducing Copper (Cu) as the dopant. It did not only increase the conductivity of 1-D ZnO but also preconcentrated the target analyte on the nanostructure surface because of the inherent electric field generated at the copper/zinc oxide heterojunction interface [99]. It was concluded that 1-D ZnO was a great active site after having been doped by Cu.

#### 1-D ZnO-Based Field Effect Transistor (FET) Biosensors

**Figure 10:**
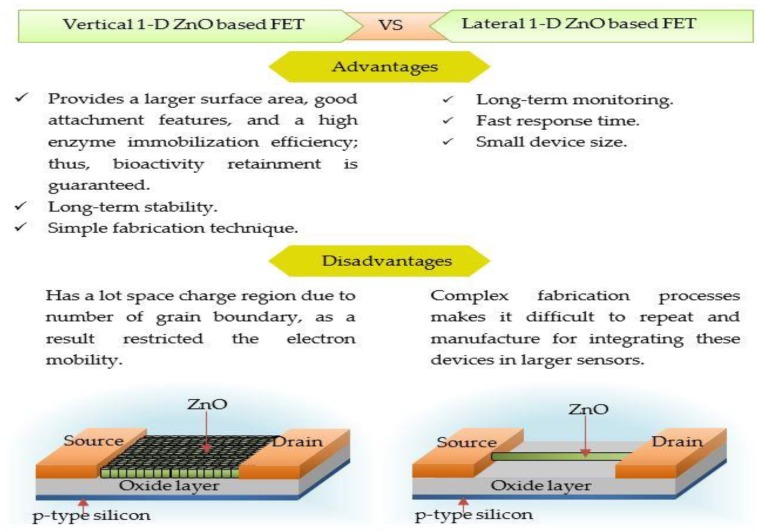
Schematic diagram of vertical and lateral 1-D ZnO-based FET biosensor with advantages and disadvantages.

Since the development of 1-D ZnO with great performances in a wide area of applications, there has been a major drive for it to be constructed in FET-based biosensors. At present, considerable efforts have been made to create a better performance of 1-D ZnO FET architecture, which can be divided into two groups: vertical 1-D and lateral 1-D ZnO FETs. Figure 10 shows the schematic diagram of these two structures with their respective advantages and disadvantages.

**Figure 11:**
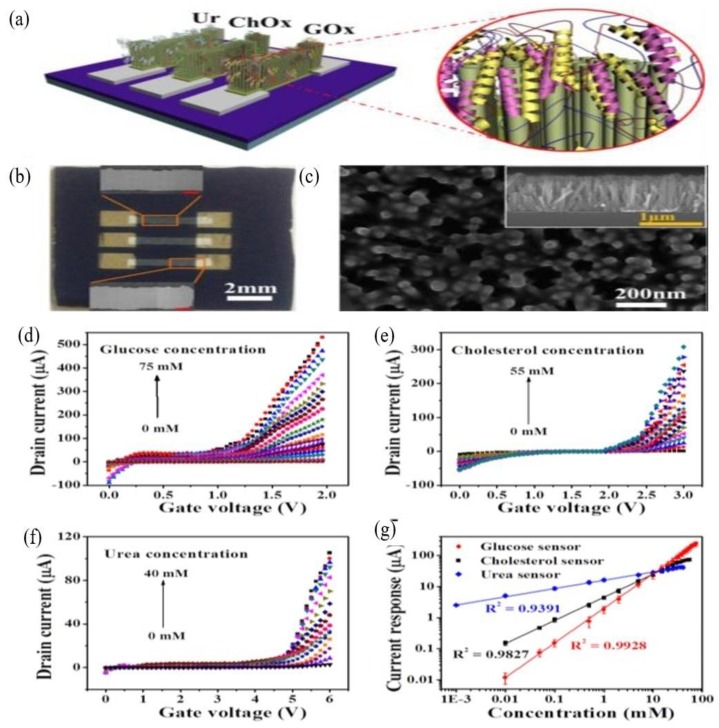
(**a**) Diagram of 1-D ZnO-based FET biosensors with immobilization of three difference enzymes. (**b**) Optical image of 1-D ZnO-based FET biosensors showing three different active channels and an FESEM image of their selected area. (**c**) FESEM top and cross-sectional (inset) images of 1-D ZnO after and before enzyme immobilization, respectively. *I_D_-V_G_* response of 1-D ZnO-based FET biosensors with increasing (**d**) glucose (0–75 mM), (**e**) cholesterol (0–55 mM), and (**f**) urea (0–40 mM) in 0.05 M PBS buffer. (**g**) Corresponding calibration plots in log scale showing linearity with a high regression coefficient. Republished with permission from Royal Society of Chemistry, from Ref. [100], Copyright 2015.

Multiple researchers have considered vertical 1-D ZnO as a potential enzymatic biosensor because of its high electron efficiency and good conduction pathways. Vertical 1-D structures are also easy to fabricate and provide long-term stability. However, it suffers from poor electron mobility due to the presence of grain boundaries along the current path from source to drain electrodes. Lateral 1-D ZnO FETs can provide long-term monitoring and faster response times. However, the complex fabrication process hampers the repeatability and manufacturability of these devices. Ahmad et al. [100] proposed a new analyte detection method for glucose, cholesterol, and urea using a FET biosensor with a vertical 1-D ZnO channel. This method’s arrays and *I_D_-V_G_* responses are shown in Figure 11a–f, respectively. Ahmad et al.’s detection method showed substantial current increases with increased analyte concentrations at room temperature. Hence, a calibration curve of the fabricated sensor current response versus analyte concentration was plotted (Figure 11g) and used for the calculation of sensitivity and linear range. The fabricated 1-D ZnO FET biosensors showed sensitivities of 32.27, 17.10, and 14.23 µA cm^−2^ mM^−1^ and detection limits of 0.07, 0.04, and 0.032 µM for glucose, cholesterol, and urea, respectively. The new FET biosensor showed a 94% response rate over 50-time measurements and 40 d of storage for each analyte. The FET sensor showed improvements in analyte detection over a number of linear ranges without sample dilution, making it suitable for clinical use.

Ma et al. [101] created a high electron mobility transistor (HEMT) that could detect lactic acid using a vertical 1-D ZnO that was doped with indium. Unlike conventional amperometric-based lactic acid sensors, HEMT biosensors do not require fixed solution reference electrodes and are capable of measuring potential between the reference electrode and nanomaterial. Ma et al.’s biosensor detected lactic acid through its drain current, which was measured through the application of the current signal. The top of the Al/GaAs/GaAs HEMT gates were coated with vertical 1-D ZnO because of its fast response time, stable detection ability at certain concentrations, and ability to efficiently immobilize lactate oxidase (LOx). The HEMT biosensor detected low lactic acid concentrations (pM) in less than 1 s. However, in another improved work, Lee et al. studied on how to fix the existence of the Fermi level pinning effect (restriction of the band alignment of the electrolyte/semiconductor junction), which lead to degradation of the sensing performances of the biosensors [102]. Under photoelectrochemical (PEC) passivation, the 1-D ZnO-based HEMT biosensor exhibited a high sensitivity of 38.9 µA/mM (higher sensitivity compares to previous work) because of the suppression of the Fermi level pinning effect.

**Figure 12:**
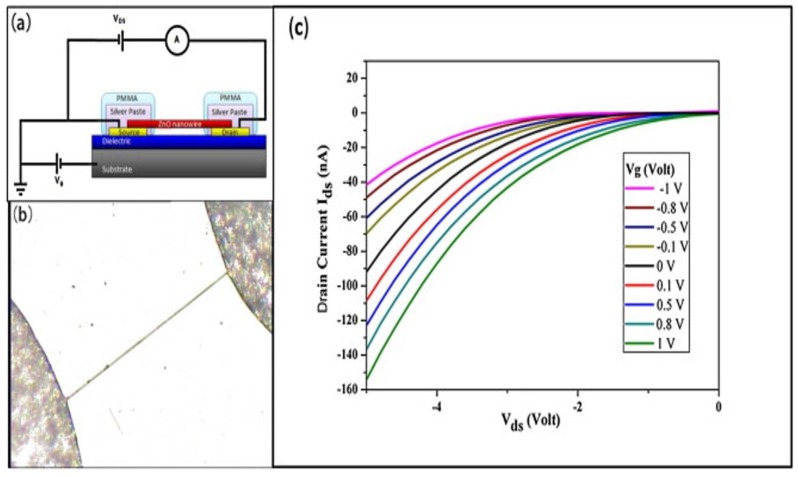
(**a**) Diagram of a single 1-D n-ZnO-based FET biosensor; (**b**) Optical image of 1-D ZnO bridging between source and drain contact; (**c**) *I_ds_* vs. *V_ds_* measurements by varying gate voltage (*V_g_*), *V_ds_* = −1 V. Reprinted from Ref. [104]. Copyright 2013, with permission from Elsevier.

Lateral 1-D ZnO-based FET biosensors possess unique characteristics compared to vertical channel sensors. There is an experimental study on lateral nanowire parameter factors based on FET biosensors toward proteins, nucleic acids, and virus detection [103]. The factors such as diameter and number of nanowires play a vital role for maximizing the sensitivity of biosensors. According to Jason et al. [103], device sensitivity decreased when the number of nanowires increased, with 4 and 7 nanowires demonstrating 35% and 82% reductions in sensitivity, respectively. This is because closely spaced nanowires in multi-nanowire FET devices compete for free analyte molecules, causing each nanowire to have a smaller charge and the overall device to have a lower sensitivity. Small nanowire diameters (60–80 nm) also exhibit 37% better sensitivity compared to large nanowire diameters (101–120 nm). Lui et al. [104] fabricated single 1-D ZnO (lateral nanowire)-based FET biosensors for uric acid detection, as shown in Figure 12. They demonstrated that uric acid can be detected in the low concentration range of 1 pM to 0.5 mM with a 14.7 nS increase in conductance. As the device had been exposed to the analyte, the conductance of the device rapidly increased, with the response time recorded to be on the order of milliseconds.

**Figure 13:**
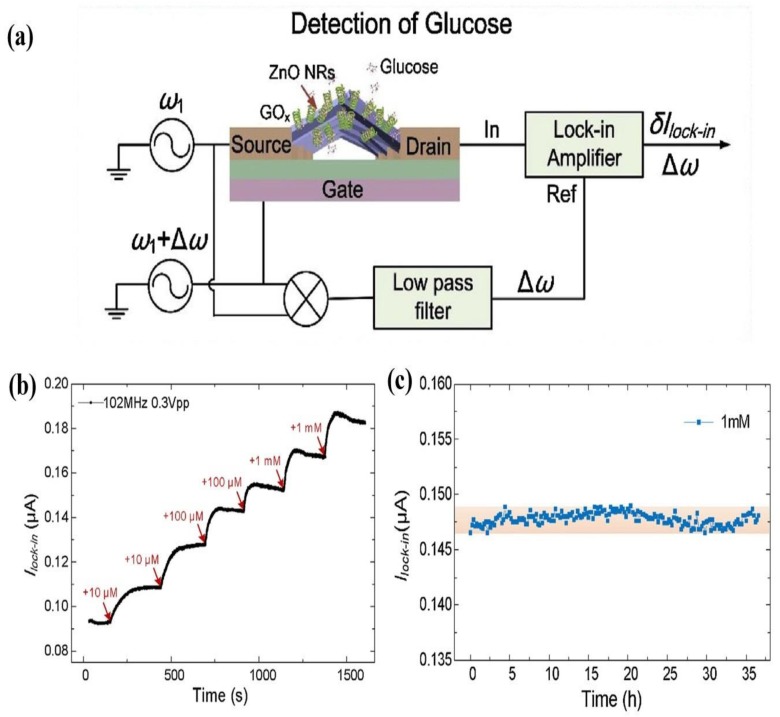
(**a**) Schematic illustration of the measuring principles of a 1-D ZnO-based FET biosensor for glucose detection. Detected lock-in currents *I*_lock-in_ (ω_1_ = 102 MHz, Δω = 30 kHz, V_sd_ = 0.3 V_pp_, V_g_ = V_pp_) over time (**b**) in PBS solution while increasing concentration (**c**) under 1 mM glucose solution at 37 °C continuously. Reprinted from Ref. [105]. Copyright 2018, with permission from Elsevier.

Recently Zong et al. [105] showed that their lateral 1-D ZnO-based FET biosensor had higher sensitivity compared to vertical 1-D ZnO biosensor works by Ahmad et al. [100], Kim et al. [94], and Wei et al. [106]. They grew the 1-D ZnO between an Au source and drain using the AC electric field assisted hydrothermal method. Their device induced a glucose concentration current response at certain frequencies by acting as a frequency mixer, as shown in Figure 13a. Their results indicated that increased glucose concentrations showed an increased lock-in current. Figure 13b shows dynamic responses of lock-in currents for 1-D ZnO-based FET biosensors from a change in glucose concentration of 0 to 2.22 mM. This resulted in the 1-D ZnO-based FET biosensor being able to monitor glucose levels over 38 h, as shown in Figure 13c. Further literature surveys about 1-D ZnO-based electrochemical biosensors research conducted since 2014 have been summarized in Table 2.

### 2.3. Two-Dimensional ZnO Nanostructures (2-D ZnO)

**Figure 14:**
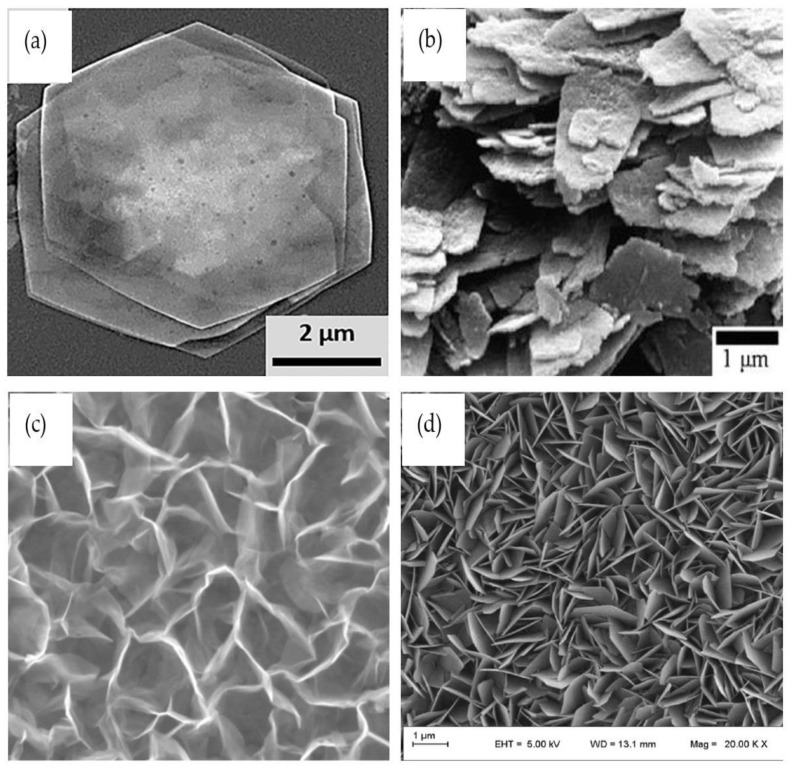
SEM image of 2-D ZnO (**a**) nanodisks/nanoflakes. Reprinted with permission from [121]. Copyright 2014 American Chemistry Society. (**b**) Nanosheets. Reprinted by permission from Springer Nature, Journals of Materials Science: Materials in Electronics [122], Copyright 2015. (**c**) Nanowalls. Adapted by permission from John Wiley and Sons from Ref. [123]. Copyright 2012. (**d**) Nanoforest. Reprinted by permission from Springer Nature [124], Copyright 2018.

In addition to the 1-D ZnO, producing other dimensional ZnO shapes has been the subject of intense research in the past few years. In this field, experiments have been conducted by focusing on the growth of nanosheets, nanoflakes, and nanowalls of ZnO on solid substrates. These morphologies are known as the 2-D group. 2-D ZnO materials are formed with Zn and O atoms stacked in such a way that they have weak layer relations (creating hexagonal films), transforming the wurtzite crystals they are based upon. Examples of a few 2-D ZnO structures are shown in Figure 14. In the flake shape of Figure 14a, the layers of nanodisks/nanoflakes horizontally cover the substrate. The same situation with nanosheets (looks like a piece of paper) is shown in Figure 14b. However, nanoflakes and nanosheets have the possibility to grow vertically and stand on a flat substrate. Usually, vertical nanosheets are known as nanowalls (Figure 14c). Vertical nanoflakes aggregated in a densely packed manner are known as nanoforests (Figure 14d).

Hydrothermal and solvothermal procedures have been utilized to synthesize the majority of 2-D ZnO structures due to the simplicity, scalability, cost, flexible substrate compatibility, and low processing temperatures of these methods. These methods dissolve ZnO precursors into an organic solvent over 3–12 h at a temperature of 75–200 °C. Previously, these methods were not successful to produce 2-D ZnO biosensing applications on a large scale. Despite the lack of reference sources, 2-D ZnO materials research has focused on limited analyte detection.

Xu and Wang [40] revealed that 1-D ZnO possess a few drawbacks in the complementary metal-oxide-semiconductor (CMOS) process for biosensors, which are small signal strength, difficulty in making electrical contacts, incompatibility with the CMOS process, and the conventional nanostructure synthesis process that generally requires a high temperature. Thus, Vabinna et al. [34] introduced highly sensitive, selective, and label-free electrochemical biosensors for cortisol detection with antibody-decorated 2-D ZnO. They created ZnO nanoflakes on a polarized (0001) plane using sonochemical methods. Anti-cab (antibody) loading and sensing performance were increased through the greater surface charge density and favorable catalytic activity of the ZnO (0001) crystal plane. Sensing parameters were measured physiologically on human saliva samples with a sensitivity of 7.74 mA/M and a lower detection limit of 1 pM, which is 100 times greater than conventional enzyme-linked immunosorbent immunoassays (ELISA). Performance was validated using the ELISA method.

2-D ZnO (nanoflakes) also had been used in uric acid [125] and glucose [126] detection. Previously, Ali et al. [125] demonstrated a simple hydrothermal fabrication for porous ZnO nanoflakes with a wall thickness of around 50–100 nm. The proposed electrochemical sensor showed an excellent sensitivity of 66 mV/decade toward uric acid elements. Furthermore, it retained up to 80% of performance after being periodically used for more than three weeks. As shown, porous ZnO nanoflakes provide a suitable environment for immobilization of enzyme molecules because of its porosity and the minor loss of enzyme during the experiment.

**Figure 15:**
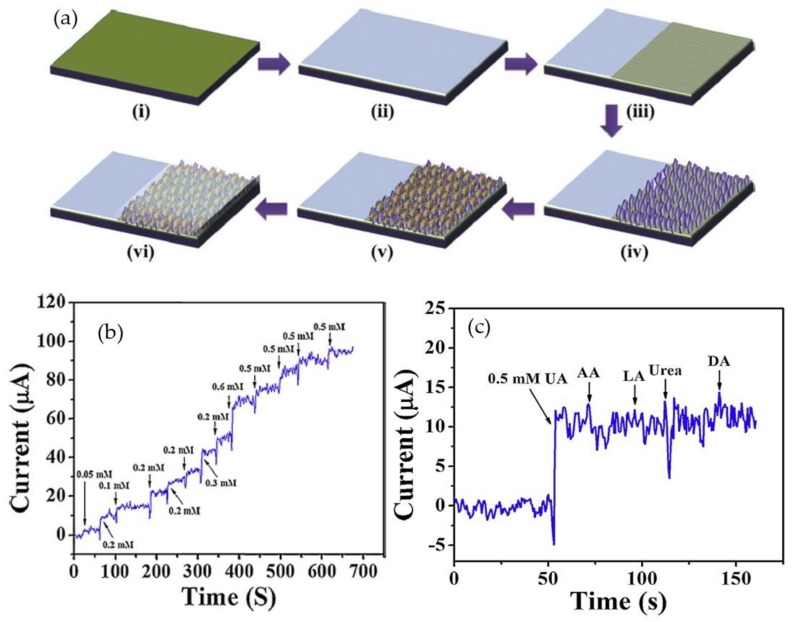
(**a**) Illustration of the fabrication process for a uric acid biosensor; (**b**) amperometric response of the biosensor toward urid acid; and (**c**) interference test of the uric acid biosensor upon addition of electroactive species. Reprinted from Ref. [127]. Copyright 2015, with permission from Elsevier.

Use of 2-D ZnO is not limited to the nanoflake morphology group. Rafiq et al. [127] reported fabrication of uric acid biosensors based on ZnO nanosheets on a silicon substrate by a one-step hydrothermal process. As shown in Figure 15a, first, they sputtered Ag metal on a Si substrate to enhance its surface adhesive behavior. Then, a seed layer was sputtered above the substrate target to promote the nucleation process. ZnO nanosheets were grown on the electrode by a low-temperature hydrothermal process. After that, uricase enzyme immobilization was applied by physical adsorption. Enzyme leaching and sample fouling were prevented using Nafion. A 50 mM current increase with a 5 s response time was seen in the amperometric response of the biosensor towards uric acid, as demonstrated in Figure 15b. The introduction of 0.25 mM of uric acid and 0.25 mM electrospecies (uric acid, ascorbic acid, lactic acid, and urea) increased the selectivity of the fabricated biosensor. Figure 15c shows interference tests of the uric acid biosensor with the addition of electroactive species. Interfering species did not obviously influence biosensor responses.

Rui et al. [128] investigated enzyme electron transfers for the one-step electrodeposition of 2-D ZnO nanosheets using a direct electron of cytochrome c (cyt.c) model. The study found that ZnO nanosheets provided a bio-compatible surface that maintained the cyt.c bioactivity and preserved its intrinsic activity towards H_2_O_2_. The enhanced electron transfer of cyt.c showed a redox formal potential of 338.2 ± 4.3 mV (vs. Ag|AgCl), which was superior to SnO_2_, SiO_2_, Nb_2_O_5_, and NaY zeolite electrodes. The results demonstrated that H_2_O_2_ biosensors possessed both excellent performance and selectivity free from anodic interferences (ascorbic acid, dopamine, and uric acid) and cathodic interference (O_2_) under 0.0 V (vs. Ag|AgCl). ZnO nanostructures that have natural biocompatibility and analytical characteristics have formed the basis for detecting extracellular H_2_O_2_ released from human hepatoma cells. This study provides a methodology for investigating direct electron transfers between proteins and nanostructured semiconductors that can serve as the basis for third-generation biosensors. This can help us to improve our understanding on H_2_O_2_ and reactive oxygen species (ROS) roles in pathology and physiology.

**Figure 16:**
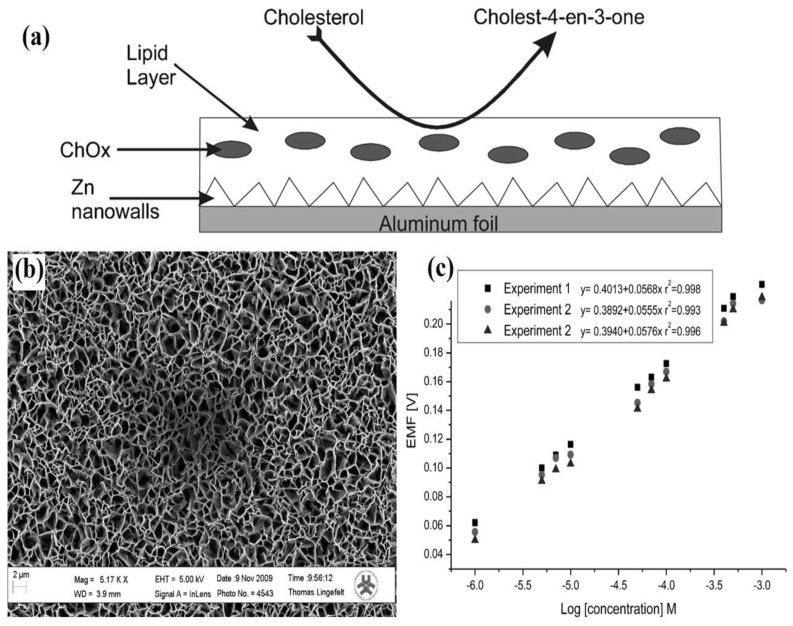
(**a**) Schematic illustration of biosensor design; (**b**) SEM image of ZnO nanowalls; and (**c**) repeated experimental electrochemical calibration curves by using a single biosensor, showing reusability of the biosensor. Adapted by permission from John Wiley and Sons from Ref. [123]. Copyright 2012.

In another work, Psychoyios et al. [123] detected cholesterol using 2-D ZnO nanowalls with stabilized polymerized films (Figure 16a,b). Cholesterol oxidase absorption capacity was improved by the high surface-area-to-volume ratio of the nanowalls and high cholesterol solubility of the lipid matrixes. The high surface-area-to-volume ratio of ZnO nanowalls allow alternate positive and negative layers to form along their nonpolar planes, aiding in the absorption of ChOx through the creation of an ideal microenvironment. The results showed a rapid response time of 5 s and a 95% steady-state voltage, which shows a quick electrochemical single transfer rate for ZnO nanowalls and cholesterol molecules. The sensitivity of the fabricated biosensor increased from 32 mV/decade to 57 mV/decade with a large, dynamic logarithmic detection range (1 × 10^−6^ to 1 × 10^−3^). Figure 16c displays three different calibration curves for the same sensor electrode and the same Ag|AgCl reference electrode. The fabricated biosensor showed excellent stability and linearity in a pH 7.4 of phosphate-buffered saline (PBS) solution. PBS was used to soak the sensor electrode after each experiment, after which it was stored at 48 °C for more than three weeks. After the sensor electrode was removed from storage, it was found to exhibit a good cholesterol response with the retention of 90% of its original activity. This preservation of enzymatic activity was due to the lipidic film environment the sensor was stored in, which increased its biocompatibility.

ZnO nanowalls-based electrochemical biosensors have also been used to monitor promyelocytic leukemia and retinoic acid receptor alpha (PML/RARA) fusion genes that can cause acute promyelocytic leukemia (PML) [129]. In this work, they deposited vertically standing ZnO nanosheets on negatively charged MoS_2_ by using electrodeposition. MoS_2_ served to provide a high electrolytic accessible surface area to adsorb Zn^2+^ during the nucleation process. Results indicated that ZnO nanowalls/MoS_2_ brought a higher hybridization efficiency than those of the other materials (ZNO/GCE and MoS_2_/GCE). The deposition time (900 s), concentration of MoS_2_ (2 g/L), reduction potentials of pulsed potentiostatic electrodeposition (−1.0 V), and concentration of zinc precursor (0.15 M) were optimized in this work. Methylene blue accumulated on the surface of the DNA-modified electrode as the external indicator, and device characteristics were measured. Results show a low detection limit of 6.6 × 10^−16^ M. Further literature surveys about the 2-D ZnO-based electrochemical biosensor research conducted since 2014 have been summarized in Table 3.

### 2.4. Three-Dimensional ZnO Nanostructures (3-D ZnO)

**Figure 17:**
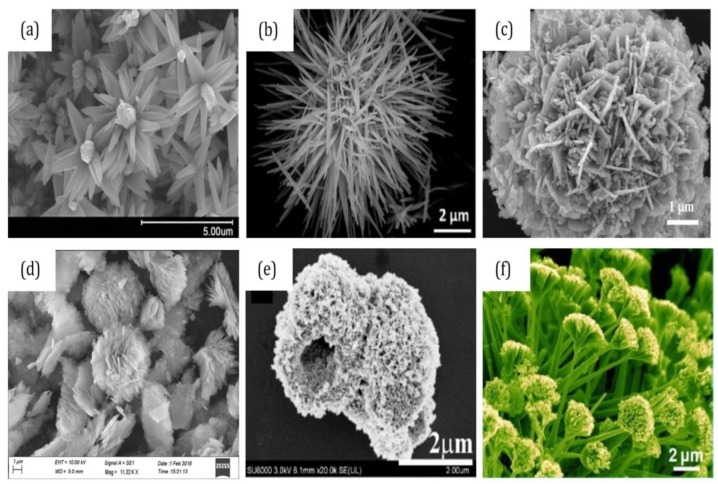
(**a**,**b**) 1-D as the building unit of 3-D ZnO [134,135]; (**c**,**d**) 2-D as the building unit of ZnO [136,137]; (**e**) hollow sphere [138]; (**f**) nanoflower grown on an alumina substrate [139]. Reproduced with permission from: (**a**) Ref. [134], Copyright 2015, Elsevier; (**b**) Ref. [135], Copyright 2018, Springer Nature, Journal of Materials Science: Materials in Electronics; (**c**) Ref. [136], Copyright 2018, Elsevier; (**d**) Ref. [137], Copyright 2018, Springer Nature, Journal of Materials Science: Materials in Electronics; (**e**) Ref. [138], Copyright 2017, Elsevier; and (**f**) Ref. [139], Copyright 2011, AIP Publishing.

ZnO nanostructures have been fabricated as three-dimensional nanostructures assembled from zero-dimensional (0-D), one-dimensional (1-D), and two-dimensional (2-D) nanostructures. 3-D nanostructures have been proven to be versatile and greatly influence the bio-recognition sensitivity. These hierarchical architectures such as nanoflowers, agglomeration of nanorods, nanospheres, and hollow spheres have attracted great interest because of their novel structures, high surface-to-volume ratio, and porosity. Figure 17 shows a myriad of 3-D ZnO nanostructures from different subunits. From the images, 3-D ZnO demonstrates various sizes and shapes. Such morphological aspects of 3-D ZnO offer potential applications in the bottom-up fabrication of biosensors, as they are easy to control and have high crystallinity with less structural defects [132]. 3-D ZnO nanostructures have been synthesized by different methods. Among them, the hydrothermal method is the least energy-consuming and also offers better control over the size of the nanostructures [133].

One of the unique characteristics of this morphological structure corresponds to its volume in geometric spaces. As Ahmad et al. [140] found, more pillar/porous 3-D nanostructures make them highly desirable as fillers for bioreceptor loading during biosensor fabrication. The fabricated amperometric biosensor excellently detected glucose in 0.1 M PBS solution between −0.20 to +0.80 V of potential range with low detection limit (50 μM), wide linear (0.05–23 mM), and high sensitivity (210.8 μA/mM cm^2^). The high sensitivity (210.8 μA/mM cm^2^) was better than previous works: 61.78 μA/mM cm^2^ using ZnO nanorods on a platinum electrode [141] and 70.2 μA/mM cm^2^ using ZnO nanofibers on a gold electrode [142].

For further advancement in glucose detection, Linxia et al. [143] demonstrated hybrid nanostructures between gold nanoparticles (AuNPs) and 3-D ZnO, which can overcome the direct electron transfer problem between some redox proteins and electrode. Additionally, the high surface area of 3-D ZnO, due to interspaces among its building unit, provided a great platform to hybridize with AuNPs. The fabricated AuNPs/3-D ZnO used with a modified glass carbon electrode (GCE) had a low detection limit (0.02 mM) in a wide linear range (1–20 mM) and showed good suitability in real, practical applications, as its relative error was less than 4% compared to hospital data.

Previously, Linxia et al. [144] proved the selectivity of hybrids between AuNP and 3-D ZnO in the electrochemical detection of dopamine (simultaneously with a uric acid element). In order to evaluate the practical ability, a biosensor was used to check the urine sample. The measured dopamine content was 2.00 × 10^−6^ M. Recoveries in the range of 94.0%–104.0% with a relative standard deviation (RSD) of 2.6%–3.8% were obtained using standard addition methods. However, a different situation occurred when Hussain et al. [145] found that silver nanoparticles (AgNP) hybridized with 3-D ZnO had a better sensor performance compared to the use of AuNP/3-D ZnO in hydrogen peroxide detection. A good amperometric response, with a linear range from 1 to 20 µM and detection limit of 2.5 µM (S/N = 3) to hydrogen peroxide, was demonstrated by the sensor. A high and reproducible sensitivity of 50.8 µA cm^−2^ µM^−1^ was also shown, with good stability and a fast response of less than 3 s.

The agglomeration of nanorods (1-D as a building unit) into 3-D ZnO is another candidate to be applied in electrochemical biosensors. Its building unit size depends on several synthesis conditions. Yue et al. [146] demonstrated the use of ammonium hydroxide and polyetherimide in hydrothermal processes to synthesize 3-D ZnO so as to control the aspect ratio of its building unit (nanorod: length and diameter). Ammonium hydroxide possesses the ability to suppress the homogeneous nucleation process of ZnO nanorods by reacting with zinc ions to form complexes and lowers the degree of supersaturation of the reaction system. To further decrease its diameter and increase the length of the 3-D ZnO building unit, polyetherimide (PEI) will play this role as it can be adsorbed on the nanostructure’s surface. As a result, the length and diameter of 3-D ZnO (1.5 µm and 200 nm, respectively) was changed to the optimum size of 2.5 µm (length) and 50 nm (diameter). This optimized 3-D ZnO was synthesized on indium-doped tin oxide electrodes to detect Levodopa and showed excellent selectivity in the presence of uric acid.

**Figure 18:**
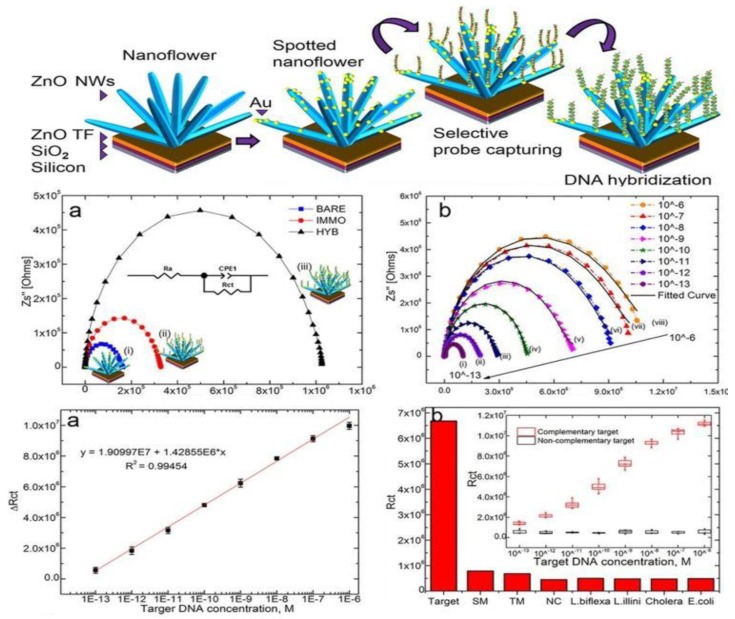
(**a**) Schematic illustration of the steps involved in the synthesis of the spotted nanoflower (NF) DNA Bioelectrode; (**b**) impedance spectra of (**i**) spotted NF, (**ii**) spotted NF/p-DNA (probe), and (**iii**) spotted NF/p-DNA/t-DNA (duplex) bioelectrode; (**c**) impedimetric response curve of spotted NF/p-DNA hybridized with different concentrations of complementary target DNA (**i**–**viii**) 10 μM to 100 fM; (**d**) the linear regression curve for different concentrations of target DNA; (**e**) bar chart of the specificity of the spotted NF bioelectrode towards mismatching and cross hybrids—the inset shows a box plot of complementary and noncomplementary target oligonucleotides hybridized to the immobilized probe oligonucleotides at different target oligonucleotide concentrations. Reprinted with permission from Nature Publishing Group, Scientific Reports, Ref. [10]. This work is licensed under a Creative Commons Attribution 4.0 International License, https://creativecommons.org/licenses/by/4.0/.

In another report, Veeradasan et al. [10] utilized a similar building unit of 3-D ZnO to fabricate DNA bioelectrodes for Leptospirosis detection (Figure 18a). A specific gene in pathogenic *Leptospira*, *hemolysis-associated protein-1* (*Hap1*), was applied as a DNA receptor on 3-D ZnO with an average length and diameter of 2–3 µm and 100 nm respectively. They used AuNP to optimize the immobilization of this DNA molecule on the 3-D ZnO surface. The charge transfer resistance (R_CT_) of the DNA bioelectrode increased rapidly upon hybridization with the target DNA, and the R_CT_ increased with increasing of complementary target DNA, as shown in Figure 18b. The fabricated DNA biosensor shows good linearity (Figure 18c,d) and a low detection limit of 100 fM. Furthermore, Figure 18e indicates this device has excellent selectivity toward different target analytes, as R_CT_ measured for Leptospirosis DNA was almost 8.5 times larger than that of single-base mismatched DNA.

Katwal et al. [147] conducted a study to use nanotubes as the building unit of 3-D ZnO. Its sensitivity towards volatile organic compounds (VOCs) was significantly affected. It was found that the Debye length and wall thickness of nanotubes plays a vital role for the electron pathway. Furthermore, the catalytic activity of platinum used as electrodes helps the sensing response because of its stable operating temperature. The development of this chemiresistive sensor is important, as the presence of this VOC in exhaled gases can be related to breast cancer. Ghanbari et al. [148] reported an important strategy to enhance thermal stability and the electron transfer rate of conducting polymer/reduced graphene oxide-based composites (PANI/RGO nanocomposite). They utilized 3-D ZnO to modify the electrocatalytic properties and sensor behavior of biosensor nanocomposites towards dopamine and uric acid detection due to strong electronic interaction between constituent materials (graphene, conducting polymer) and ZnO. The 3-D ZnO were deposited by using a potentiostatic method at −0.7 V for 2 min. In ZnO crystals, Zn structures were oxidized by exposing them to a 0.1 M sodium hydroxide solution that cycled between −0.5 to 1.0 V. The mixture solution showed a linear response between concentration ranges of 0.1–90 µM and 90–1000 µM and a DA detection limit of 0.017 µM (S/N = 3). The mixture solution also showed a linear range between 0.5–90 µM and 100–1000 µM with a UA detection limit of 0.12 µM (S/N = 3) in addition to the coexistence of 1000 µM AA. The coasting of the PANI/RGO nanocomposite with 3-D ZnO increased its active surface area, strong adsorptive capability, and specific interaction abilities, increasing its DA and UA electrochemical platforms. This study proved that 3-D ZnO has promising morphological features for constructing electrochemical biosensors. Uses of 3-D ZnO with graphene nanocomposites are not limited to dopamine and uric acid detection. Junwei et al. [149] fabricated 3-D ZnO/reduced graphene oxide-based nanocomposites on glassy carbon electrodes for the detection of hydrazine. The fabricated electrochemical biosensor showed excellent sensitivity, with a detection limit of 0.8 µM and a linear detection range from 1.0 to 33.5 µM. Table 4 summarizes the 3-D ZnO-based electrochemical biosensor research conducted since 2014.

## 3. Conclusions

Biosensor development is becoming increasingly popular as it intersects the biological and engineering sciences. The maturity of semiconductor technology has encouraged the rapid adoption of new sensors using nanotechnology. This has created a new field of nano-biosensing, which exemplifies how engineering sciences, physics, chemistry, and biology intersect at the nanometer scale. The introduction of nano-transducers in sensing technology is making the traditional separation of transducers and bioreceptors obsolete. Nano-biosensing as the early point-of-care detection of disease markers at affordable prices is becoming critical for healthcare in developing countries. This is evidenced by the distributed monitoring of diabetes mellitus underlying both the research and commercial development of glucose sensors. Nanotechnology has the potential to develop integrated electrochemical sensors with high throughput through the creation of extremely sensitive sensors such as nanowires, which has the potential of reaching a sensitivity down to a single molecule.

ZnO nanostructures as active sites show significant biosensor potential. This is because ZnO nanostructures have morphologies with large surface areas, allowing for the fabrication of devices with diverse structures. In addition, the high IEP of ZnO nanostructures encourages electrostatic adsorption of enzymes and the retention of enzyme activity through favorable microenvironments. ZnO nanostructures that exhibit high crystallinity create direct electron conduction tunnels between enzyme sites and electrode surfaces.

This study investigated ZnO structures with different dimensions, especially electrochemical biosensors and their respective sensing performances. The potential of 0-D, 1-D, 2-D, and 3-D ZnO nanostructures as biosensors was discussed. 0-D ZnO nanostructures had been used widely due to its ease of fabrication before 1-D, 2-D, and 3-D nanostructures were developed. However, the focus turned towards 1-D ZnO because of the issues with low mobility faced in 0-D nanostructures. However, growth of the nanotechnology field nowadays has allowed the different exploration of ZnO quantum dots. Therefore, the improvement of 0-D ZnO-based electrochemical biosensor nanostructures is positive.

In addition, the vertical and lateral 1-D ZnO-based FET biosensor nanostructures are also proven to enhance the performance of the biosensor. However, the current challenge is to produce large quantities of vertical 1-D ZnO nanostructures with well-controlled dimensions. Similarly, lateral 1-D ZnO nanostructures are not easy to align between source and drain in order to get a consistent sensor reading. Meanwhile, 2-D ZnO nanostructures exhibit the highest surface-to-volume ratio and specific facet (0001) that offers an efficient immobilization loading process. Through the experiment test where 2-D ZnO nanostructures were used, the result showed that it is possible to tune the sensing performance. Furthermore, the ideal case is to create a planar environment with minimal scattering sites to gain the maximum mobility. All the above makes 2-D ZnO nanostructures extremely promising for the future. Similarly, porosity of 3-D ZnO nanostructures has made it attractive in amperometric-, potentiometric-, and impedimetric-based biosensors. However, use of this 3-D ZnO nanostructure as an active channel in FET-based biosensors has not yet received attention.

Creation of smaller, faster, and cheaper sensors is being driven by biosensor research, which one day may result in the integration of electronic and biological systems. A combination of quantum mechanics, surface physics, biology, bioengineering, and electrical engineering may be required to create highly sensitive, highly specific, multianalysis, and nanoscale biosensors and bioelectronics, which would greatly benefit early diagnostics and health care.

## Figures and Tables

**Table 1:** Different techniques of 0-D ZnO-based electrochemical biosensors for various analyte detections, since 2014.

Morphology	Technique	Bio-Receptor	Doping/Hybrid	Analyte	Sensing Performance: Sensitivity (S), Limit of Detection (LOD), Linear Range (LR), Time Response (T)
Nanoparticle [66]	Impedimetric	Nonenzymatic	Cu-doped	Glucose	S: N/A LOD: 10^−9^ M LR: 10^−9^–10^−5^ M T: N/A
Nanoparticle [52]	Amperometric	Nonenzymatic	N/A	Glucose	S: 631.30 µA mM^−1^ cm^−2^ LOD: 0.043 µM LR: 1–8.6 mM T: 4 s
Nanoparticle [81]	Amperometric	GOx	Nitrogen-doped carbon sheets hybrid	Glucose	S: 231.7 µA mM^−1^ cm^−2^ LOD: 6.3 µM LR: 0.2–12 mM T: 3 s
Nanoparticle [82]	Voltammetric	GOx	Graphene–carbon nanotube (CNT) hybrid	Glucose	S: 5.362 µA mM^−1^ cm^−2^ LOD: 4.5 µM LR: 10 µM to 6.5 mM T: N/A
Nanoparticle [83]	Voltammetric	Enzymatic	Multiwall CNT hybrid	Levodopa	S: N/A LOD: 0.08 µmol L^−1^ LR: 5.0–500.0 µmol L^−1^ T: N/A
Nanoparticle [84]	Voltammetric	Probe DNA	Platinum and palladium hybrid	Dengue	S: N/A LOD: 43 µM LR: 1 µM–100 µM T: N/A
Nanoparticle [85]	Amperometric	ChEt & ChOx	N/A	Cholesterol	S: 190 µA mM^−1^ cm^−2^ LOD: N/A LR: 0.5–12 mM T: 5 s
Nanoparticle [86]	Voltammetric	Nonenzymatic	Multiwall CNT hybrid	Citalopram	S: N/A LOD: 0.005 µM LR: 0.012–1.54 µM T: N/A
Quantum dot [87]	Amperometric	Nonenzymatic	Graphene oxide/tyrosinase hybrid	Hydroxylated polychlorobiphenyls	S: N/A LOD: 0.15 µM LR: N/A T: N/A
Quantum dot [88]	Amperometric	Nonenzymatic	Reduced graphene oxide and multiwall CNT hybrid	Ascorbic acid	S: 36.12 µA mM^−1^ cm^−2^ LOD: 3.42 µM LR: 10–600 µM T: 2 s
Quantum dot [89]	Voltammetric	Starved algal cells	Coated by SiO_2_	Acephate	S: N/A LOD: 1.0 × 10^−12^ M LR: 10^−11^–10^−3^ M T: N/A
Quantum dot [90]	Voltammetric	Enzymatic	Coated by nafion layer	Uric acid	S: 4.0 µA mM^−1^ cm^−2^ LOD: 22.97 µM LR: 1 mM–10 mM T: N/A

**Table 2:** Different techniques of 1-D ZnO-based electrochemical biosensors for various analyte detections, since 2014.

Morphology	Technique	Bio-Receptor	Doping/Hybrid	Analyte	Sensing Performance Sensitivity (S), Limit of Detection (LOD), Linear Range (LR), Time Response (T)
Nanorod [107]	Impedimetric	Nonenzymatic	N/A	Cholesterol	S: 4.2 µm mM^−1^ cm^−2^ LOD: 1.78 mM LR: 1–9 mM T: N/A
Nanotube [98]	Amperometric	Enzymatic ChOx	N/A	Cholesterol	S: 79.40 µA mM^−1^cm^−2^ LOD: 0.5 nM (S/N = 3) LR: 1.0 µm–13.0 mM T: 2 s
Nanotube [108]	Amperometric	Nonenzymatic	Au nanoparticles hybrid	Hydrogen peroxide	S: 1336.7 µm mM^−1^ cm^−2^ LOD: N/A LR: 1 µM–3.0 mM T: N/A
Nanowire [109]	Amperometric	Nonenzymatic	CO_3_O_4_/NiCo_2_O_4_/Ni foam hybrid	Hydrogen peroxide	S: 0.388 mA mM^−1^ cm^−2^ LOD: 0.163 µM (S/N = 3) LR: 0.2 µM–2.4 mM T: 5 s
Nanowire [110]	Amperometric	Enzymatic	N/A	L-lactic acid	S: 15.6 µA mM^−1^ cm^−2^ LOD: 12 µM LR: 12 µM–1.2 mM T: N/A
Nanowire [111]	Voltammetric	Nonenzymatic	Graphene/graphene foam	Folic acid	S: 0.18 µm mM^−1^ LOD: 1 µM LR: 0–60 µM T: N/A
Nanowire [112]	Voltammetric	Nonenzymatic	Graphene foam hybrid	Levodopa	S: 3.15 µA µM^−1^ LOD: 50 nM LR: 0.05–20 µM T: N/A
Nanorod [113]	Potentiometric	N/A	Reduced graphene oxide hybrid	Uric acid	LOD: 0.312 µM (S/N = 3) LR: 1–800 µM
Nanorod [114]	Amperometric	Uricase	N/A	Uric acid	S: 239.67 µA mM^−1^ cm^−2^ LOD: 5 nM LR: 0.01–4.56 mM T: 3 s
Nanorod [115]	Voltammetric	Urease	Ag hybrid	Urea	S: 41.64 µA mM^−1^ cm^−2^ LOD: 10 µM LR: 0.001–24.0 mM
Nanotube [8]	Amperometric	GOx	N/A	Glucose	S: 2.63 µA mM^−1^ cm^−2^ LOD: 8 µM (S/N = 3) LR: 0–6.5 mM
Nanorod [116]	Amperometric	Nonenzymatic	CuO hybrid	Glucose	S: 2961.7 µA mM^−1^ cm^−2^ LOD: 0.40 µM LR: Up to 8.45 mM T: <2 s
Nanorod [117]	Voltammetric	Nonenzymatic	Copper nanoparticles hybrid	Glucose	S: 609.6 µA mM^−1^ LOD: 0.03 µM (S/N = 3) LR: 5 µM–1.1 mM
Nanorod [6]	Amperometric	GOx	N/A	Glucose	S: 10.911 mA mM^−1^ cm^−2^ LOD: 0.22 µM LR: 0.6–1.4 mM T: 3 s
Nanorod [118]	FET	Nonenzymatic	Fe_2_O_3_ hybrid	Glucose	S: 105.75 µm mM^−1^ cm^−2^ LOD: 12 µM (S/N = 3) LR: 0.05–18 mM
Nanorod [119]	FET	Nonenzymatic	NiO quantum dots hybrid	Glucose	S: 13.14 µA mM^−1^ cm^−2^ LOD: 26 µM LR: 0.00–10 mM
Nanorod [105]	FET	GOx	N/A	Glucose	S: 1.6 mA mM^−1^ cm^−2^ LOD: 1 µM
Nanorod [120]	Liquid gate FET	Antibody	N/A	Hepatitis B	LOD: 20 aM LR: 20 aM to 1 pM

**Table 3:** Different techniques of 2-D ZnO-based electrochemical biosensors for various analyte detections, since 2014.

Morphology	Technique	Bio-Receptor	Doping/Hybrid	Analyte	Sensing Performance Sensitivity (S), Limit of Detection (LOD), Linear Range (LR), Time Response (T)
Nanowall [130]	Amperometric	Nonenzymatic	N/A	Glucose	S: 700.6 1 µm mM^−1^ cm^−2^ LOD: 1 µm (S/N = 3) LR: 1 µm to 19.2 mM T: N/A
				Hydrazine	S: 660.2 µm mM^−1^ cm^−2^ LOD: 0.5 µM (S/N = 3) LR: 0.5 µM to 14.2 mM T: N/A
Nanosheet [128]	Amperometric	Uricase	Ag hybrid	Uric acid	S: 129.81 µA mM^−1^ cm^−2^ LOD: 0.019 µM LR: 0.05–2.0 mM T: 5 s
Nanosheet [24]	Amperometric	Nonenzymatic	Graphitic carbon nitride	Hydrogen peroxide	S: 540.8 µA mM^−1^ cm^−2^ LOD: 1.7 µM LR: 0.05–14.15 mM T: N/A
Nanosheet [129]	Voltammetric	DNA	MoS_2_ hybrid	PMA/RARA fusion gene	S: N/A LOD: 6.6 × 10^−16^ M LR: 1.0 × 10^−15^ M–1.0 × 10^−6^ M T: N/A
Nanoflake [34]	Amperometric	Anti-cortisol antibody	Bovine serum albumin and Au for immobilizing and hybrid, respectively	Cortisol	S: 7.74 µA/M LOD: 1 pM LR: N/A T: N/A
Nanoporous [131]	Impedimetric	Urease	N/A	Urea	S: 0.637 kΩ mM^−1^ LOD: 0.40 mM LR: 0.83–23.2 mM T: N/A

**Table 4:** Different techniques of 3-D ZnO-based electrochemical biosensors for various analyte detections, since 2014.

Morphology	Technique	Bio-Receptor	Doping/Hybrid	Analyte	Sensing Performance: Sensitivity (S), Limit of Detection (LOD), Linear Range (LR), Time Response (T)
Spherical lamellar [143]	Voltammetric	GOx	Au nanoparticles hybrid	Glucose	S: 1.409 µA mM^−1^ (S/N = 3) LOD: 0.02 mM LR: 1–20 mM
Spherical nanosheet [144]	Amperometric	Nonenzymatic	Au nanoparticles hybrid	Dopamine	LOD: 0.02 µM LR: 0.1–300 µM
Nanoflower [148]	Voltammetric	Nonenzymatic	Polyaniline/reduced graphene oxide hybrid	Dopamine	LOD: 0.8 nM (S/N = 3) LR: 0.001–1 µM and 1–1000 µM
				Uric acid	LOD: 0.042 µM (S/N = 3) LR: 0.1–100 µM and 100–1000 µM
Spherical nanosheet [150]	Voltammetric	Nonenzymatic	Graphene foam hybrid	Levodopa	S: 0.66 µA µM^−1^ LOD: 1 µM LR: 1–75 µM
Nanoflower [135]	Voltammetric	Nonenzymatic	Graphene hybrid	Levodopa	S: 0.32 µA µM^−1^ LOD: 1 µM LR: 1–60 µM
Nanoflower [10]	Impedimetric	DNA	Au nanoparticles hybrid	Leptospira	LOD: 100 fM LR: 10^−6^–10^−13^ M
Nanoflower [14]	Voltammetric and impedimetric	DNA	N/A	Bacterial meningitis	S: 168.64 µA ng^−1^ µL cm^−2^ LOD: 5 ng µL^−1^ LR: 5–240 ng µL^−1^
